# Trans-Catheter Valve-in-Valve Implantation for the Treatment of Aortic Bioprosthetic Valve Failure

**DOI:** 10.3390/jcm11020344

**Published:** 2022-01-11

**Authors:** Andrea Buono, Diego Maffeo, Giovanni Troise, Francesco Donatelli, Maurizio Tespili, Alfonso Ielasi

**Affiliations:** 1 Interventional Cardiology Unit, Fondazione Poliambulanza, 25124 Brescia, Italy; andrea.buono@poliambulanza.it (A.B.); diego.maffeo@poliambulanza.it (D.M.); 2 Cardiac Surgery Unit, Cardiovascular Department, Hospital Poliambulanza Foundation, 25124 Brescia, Italy; giovanni.troise@poliambulanza.it; 3 Department of Cardiothoracic Center, Istituto Clinico Sant’Ambrogio, University of Milan, 20149 Milan, Italy; francesco.donatelli@unimi.it; 4 Clinical and Interventional Cardiology Unit, Istituto Clinico Sant’Ambrogio, 20149 Milan, Italy; tespili@katamail.com

**Keywords:** valve-in-valve, TAVR, bioprosthetic valve failure, structural valve degeneration

## Abstract

Aortic valve-in-valve (ViV) procedure is a valid treatment option for patients affected by bioprosthetic heart valve (BHV) degeneration. However, ViV implantation is technically more challenging compared to native trans-catheter aortic valve replacement (TAVR). A deep knowledge of the mechanism and features of the failed BHV is pivotal to plan an adequate procedure. Multimodal imaging is fundamental in the diagnostic and pre-procedural phases. The main challenges associated with ViV TAVR consist of a higher risk of coronary obstruction, severe post-procedural patient-prosthesis mismatch, and a difficult coronary re-access. In this review, we describe the principles of ViV TAVR.

## 1. Introduction

In the last years, implantation of bioprosthetic heart valves (BHVs) is increasingly becoming the treatment choice in patients requiring surgical aortic valve replacement. At the same time, trans-catheter aortic valve replacement (TAVR) has been defined as the preferred mode of intervention for patients aged ≥ 75 years, independently from their surgical risk [1,2]. Despite engineering refinements, BHVs are prone to an unavoidable degeneration with the onset of structural valve degeneration (SVD) generally occurring seven to eight years after implantation [3]. Moreover, data concerning the very long-term durability of trans-catheter heart valves (THVs) are still missing [4]. Whatever the type of BHV implanted, surgical re-operative valve replacement of a failed bioprosthetic valve carries a higher risk of morbidity and mortality compared with the initial valve replacement [5]. For this reason, the procedure of valve-in-valve (ViV) TAVR has been established as a valid therapeutic option in this scenario, and the number of such procedures is inevitably estimated to exponentially increase in the next years. Despite ViV TAVR being considered a safe and effective procedure, it is more technically challenging when compared to TAVR for native aortic valve stenosis. The main reasons are represented by the bulky presence of the failed BHV and the subsequent potential mechanical complications [6]. In this review, we point out the practical aspects connected to ViV planning and performance.

## 2. Safety and Efficacy of ViV TAVR

Large registries demonstrated that ViV TAVR can be safely performed in patients at increased surgical risk [7,8,9]. Predictable advantages of ViV TAVR consist of its short-term safety. In fact, 30-day overall mortality was 7.6% in the Valve-in-Valve International Data Registry (VIVID), enrolling 459 patients [7], 2.1% in the Society of Thoracic Surgeons/American College of Cardiology Transcatheter Valve Therapies Registry enrolling 1150 patients [8], and as low as 0.7% of 365 patients in the continued access PARTNER 2 ViV Registry [9]. However, randomized controlled trials (RCTs) comparing ViV TAVR and surgical redo are not available and most of the direct comparison data came from propensity score analyses. Spaziano et al. reported similar 30-day and 1-year mortality, stroke, renal failure, and pacemaker implantation rates between treatments, with lower gradients with surgery and shorter hospital length of stay with ViV TAVR [10]. In 262 patients, selected on the basis of a 1:1 propensity-score match, Tam et al. reported lower early mortality, pacemaker implantation, and blood transfusion rates for ViV TAVR, in association with a shorter length of stay and a higher 5-year survival [11]. Similar data come from a large U.S. study, enrolling more than 4000 patients [12]. Further confirmations, that ViV TAVR is associated with better short-term outcomes than surgical redo, have been recently emerged from a French Registry comparing 717 matched patients for each treatment: lower 30-day rates of the composite of all-cause mortality, all-cause stroke, myocardial infarction (MI), and major or life-threatening bleeding have been reported in the trans-catheter cohort, whereas major cardiovascular outcomes did not differ between the two treatments during long-term follow-up [13]. A meta-analysis, including 12 publications and more than 16,000 patients proved the lower incidence of post-operative complications and better early survival of ViV TAVR, at the cost of a higher rate of MI and severe patient-prosthesis mismatch (PPM) [14]. Less evidence is available concerning the long-term safety and efficacy of ViV TAVR. The 3-year follow-up of the PARTNER 2 trial showed that TAVR for BHV failure was associated with favorable survival, sustained improved hemodynamic status and excellent functional and quality-of-life outcomes [15]. Analogous findings emerged from the CoreValve U.S. Expanded Use Study [16]: self-expanding TAVR in patients with failed surgical BHV at extreme risk for surgery was associated with durable hemodynamics and excellent clinical outcomes at 3 years. Nevertheless, data on very long-term outcomes after aortic ViV are scarce. In a retrospective study [17], the estimated survival at 8-year was 38.0%, with a median survival of 6.2 years: independent predictors of decreased patient survival were small BHV size, high patient’s age, low baseline left ventricular ejection fraction, other than transfemoral access and the presence of diabetes mellitus. Although ViV TAVR is now recognized as a good alternative to redo surgery in high-risk patients with failed surgical BHV, there are few data concerning ViV within failed THVs. In this context, Landes et al. [18] provided robust observational data to support the use of redo TAVR as a primary strategy for the treatment of degenerated THVs, reporting low peri-procedural complication rates in association with satisfying 30-day and 1-year survival (94.6% and 98.5% and 83.6% and 88.3% for patients presenting with respectively early and late THV dysfunction). Moreover, in propensity score-matched cohorts of TAVR-in-THV versus TAVR-in-surgical BHV patients, TAVR-in-THV was associated with higher procedural success and similar procedural safety and mortality [19]. An overview of the available evidence is reported in Table 1.

## 3. Type of Aortic THVs and Surgical BHVs

**Figure 1:**
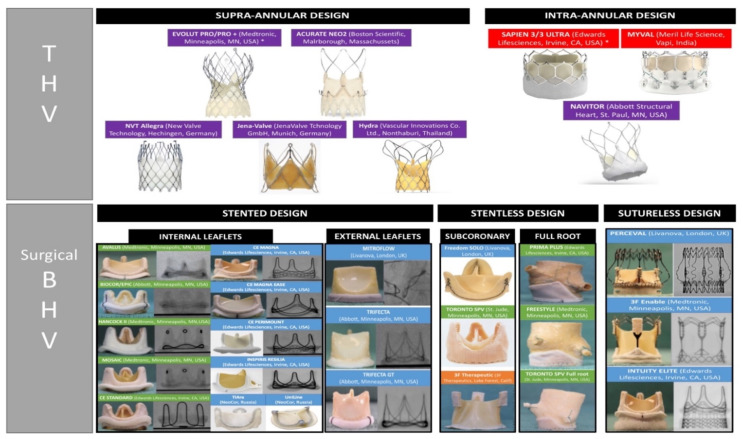
**Available portfolio of THVs and surgical BHVs** regrouped according to their main features: in purple, devices with self-expanding design; in red, devices with balloon-expandable design; in green, devices made by porcine tissue; in blue, devices made by bovine pericardial tissue; in orange, devices made by equine pericardial tissue. BHV: bioprosthetic heart valve; THV: trans-catheter heart valve. *CE mark for valve-in-valve use.

Figure 1 depicts the current portfolio of available THVs and surgical BHVs, classified according to the main device features. Concerning THVs, beyond the mode of delivery [self-expanding (SE) vs. balloon-expandable (BE)], a relevant characteristic to be mentioned is the supra- or intra-annular design. This aspect is crucial especially for a proper THV selection during ViV TAVR. Likewise, several surgical BHVs, with different features, are available on the market. Briefly, surgical BHVs are classified according to the type of leaflet tissue (porcine vs. bovine) and according to the frame design (stented, stentless, or sutureless). In detail, prosthetic leaflets can be mounted internally or externally to the BHV frame. On one hand, externally mounted leaflets allow to obtain a larger valve effective orifice area (EOA), however, on the other, increase the risk of coronary obstruction during the ViV TAVR. Among stented BHVs, another difference concerns the possibility to fracture the stent, with some BHVs in which balloon valve fracturing (BVF) is feasible, others that can undergo balloon valve remodeling (BVR), and the remaining valves that cannot undergo neither BVF nor BVR. In Table 2 we reported the feasibility of BVF/BVR in the different BHVs.

The design of stentless prostheses is intended to achieve a more physiological flow pattern and a superior hemodynamic in comparison to stented valves. Most of the stentless BHVs partially or fully replace the aortic root and can be implanted by several surgical techniques: complete or modified subcoronary, root inclusion, and full root. The full root technique is accompanied by the lowest rate of PPM [20] at the cost of an increased risk of coronary obstruction during ViV TAVR. Lastly, sutureless devices are BHVs implanted in an open surgical fashion but require few or no sutures, allowing shortened cardiopulmonary bypass and cross-clamp times [21]. It is important to note that stentless and sutureless BHVs cannot undergo BVF.

## 4. Mode of BHV Failure

**Figure 2:**
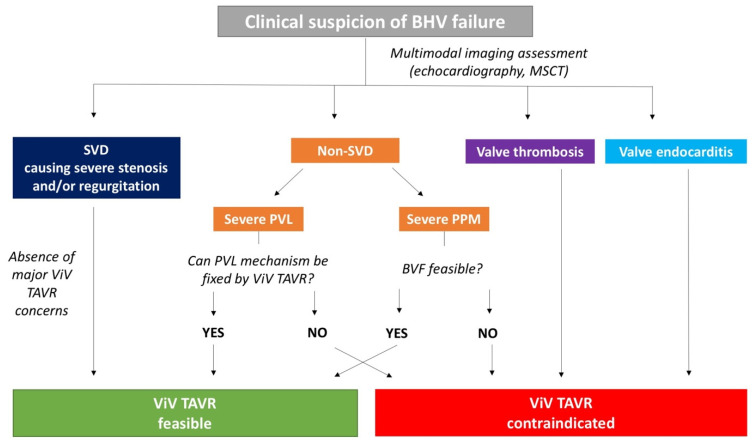
**Practical diagnostic and therapeutic algorithm in case of BHV failure suspicion**. BHV: bioprosthetic heart valve; BVF: balloon valve fracturing; MSCT: multi-slice computed tomography; PPM: patient-prosthesis mismatch; PVL: paravalvular leak; SVD: structural valve deterioration; TAVR: trans-catheter aortic valve replacement; ViV: valve-in-valve.

According to the Valve Academic Research Consortium (VARC)-3 criteria, four main mechanisms of BHV failure have been identified: SVD, non-SVD, valve thrombosis, and valve endocarditis [22]. SVD is defined as intrinsic permanent changes in BHV, including wear and tear, leaflet disruption, flail leaflet, leaflet fibrosis, and/or calcification and strut fracture, resulting in stenosis and/or regurgitation. Indeed, non-SVD encompasses paravalvular leak (PVL) and PPM. A scale of SVD has been proposed by the European Association of Percutaneous Cardiovascular Interventions endorsed by the European Society of Cardiology (ESC) and the European Association for Cardio-Thoracic Surgery [23] (Table 3). The precise diagnosis of the failure mechanism is of utmost importance to plan the proper treatment. In fact, if patients affected from SVD are suitable for ViV TAVR, in most of the non-SVD cases trans-catheter correction is not indicated and is counterproductive. If PVL is the failure mode, understanding the underlying mechanism is pivotal to predicting the ViV usefulness [24]. A practical diagnostic and therapeutic algorithm is shown in Figure 2. In general, bovine pericardial BHV is more prone to stenosis, whereas porcine leaflets tend to fail more commonly by regurgitation [6]. If THVs and surgical BHVs have similar long-term durability is still an open issue. THV leaflets are thinner, subjected to higher stresses and strain, and require crimping [25]. Despite these aspects can predict a faster deterioration, THVs have less PPM and larger mean areas. Taken together, current data show that THVs perform at least similarly to surgical BHV at five to six years. Inevitably, the occurrence of PVL is still considered the main issue related to the use of THVs than surgical replacement [26].

## 5. The Role of Pre-Procedural Multimodal Imaging

**Figure 3:**
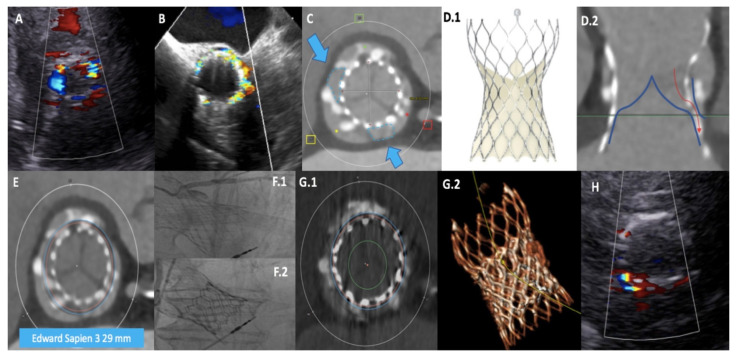
**Multimodal imaging approach to assess the feasibility of ViV TAVR for PVL correction**. A patient, with previous Evolut R 34 mm (Medtronic) implantation, presented with severe paravalvular leak (PVL) at transthoracic (**A**) and transesophageal (**B**) echocardiography, due to low device implantation. Multi-slice computed tomography (MSCT) confirmed the PVL mechanism, showing incomplete native annulus sealing by the narrow part of the trans-catheter (THV) waist (in (**C**) blue arrows indicate the two gaps); moreover, also the internal THV skirt was too low and unable to properly work (**D.1**,**D.2**). MSCT allowed a simulation of ViV TAVR using a balloon-expandable Sapien 3 29 mm (Edwards, blue circle in (**E**)), able to stretch the self-expanding device frame in order to correctly seal the native annulus. Angiographic evidence of pre-ViV TAVR PVL with confirmation of previous low THV implantation (**F.1**) and final result (**F.2**). Post-procedural MSCT showed a proper PVL mechanism correction (**G.1**,**G.2**), with only mild residual PVL at pre-discharge echocardiographic assessment (**H**). TAVR: trans-catheter aortic valve replacement; ViV: valve-in-valve.

Pre-procedural multimodal imaging is pivotal when ViV TAVR is planned. Echocardiography and multi-slice computed tomography (MSCT) are the cornerstones in both diagnostic and pre-procedural phases. Transthoracic and transoesophageal echocardiography are able to differentiate SVD from non-SVD. Abnormal leaflet structure and motion are typical of BHV SVD, thrombosis, and/or endocarditis, whereas PPM is characterized by normal valve structure. Identification of high transvalvular gradients (mean gradient > 20 mmHg, velocity peak > 3 m/s) poses the question of differential diagnosis between PPM and SVD: a marked reduction of doppler velocity index (≤0.25) in association with reduction of effective orifice area (EOA; <1 cm^2^) denotes SVD, whereas normal EOA value (with reduced indexed EOA) is consistent with PPM diagnosis. Moreover, PPM is characterized by a smaller increase in mean gradient during follow-up compared to SVD. Pressure recovery is an underestimated reason for the potential overestimation of gradients using echocardiography, especially in patients with small ascending aortic dimensions. Nevertheless, the original validation studies found highly significant correlations between simultaneous echo-Doppler and catheterization systolic gradients also in patients with surgical prosthetic aortic stenosis [27]. Due to the paucity of data on this topic in the context of THV SVD, there should be caution in relying on echo-Doppler alone to diagnose true THV obstruction: it is always important to compare with the Doppler velocity values after the procedure to determine whether there is a significant change with time. Echocardiography is the gold standard also to assess the entity, localization and mechanism of PVL, addressing the feasibility of ViV TAVR to correct the defect. An example of multimodal imaging used to guide the indication of ViV TAVR to correct a PVL is shown in Figure 3. Subclinical and clinical leaflet thrombosis are well identified at MSCT as hypo-attenuated thickening and hypo-attenuation affecting motion as hallmarks [28]. In the pre-procedural phase, MSCT is mandatory to depict the main anatomical characteristics and to measure native and BHV dimensions. In fact, beyond the standard analysis of ileo-femoral access, ascending aorta, and aortic root, MSCT proves priceless information about the virtual-to-coronary (VTC) and virtual-to-sinotubular junction (VTSTJ) distances, needed to estimate the risk of coronary obstruction when the BHV posts extend above the coronary artery orifice or the sinotubular junction (STJ), respectively. Inner stent and true inner diameter (ID) can be reproducibly measured at MSCT and predict the occurrence of potential post-procedural mismatch.

## 6. Selection of THV Type

There are no unique recommendations concerning the type of THV that should be selected for ViV TAVR. The choice should be conducted on the basis of multi-parametric analysis. In general, is important to keep in mind that BE-THVs have an intra-annular design, whereas SE-THVs can present prosthetic leaflets in a supra-annular or intra-annular position. Supra-annular THVs have been associated with a lower incidence of PPM after ViV [29] and can represent the preferred choice especially in smaller failed BHV (true ID ≤23 mm). However, if BVF is feasible, BE-THVs can be considered a valid option. BE-THVs are the first choice in case of sutureless BHV SVD presenting with pure aortic regurgitation or in case of future predictable need of coronary re-access.

The valve-in-valve app (the Aortic Valve in Valve app, Dr. Bapat and UBQO Ltd.) can help in planning and performing the procedure with a quick and easy reference guide to the anatomy, dimensions, and design features of available surgical BHVs and THVs, including their fluoroscopic appearances [30]. Lastly, only Evolut (Medtronic, Minneapolis, MN, US) and Sapien (Edwards Lifesciences, Irvine, CA, US) THVs have so far obtained the CE mark approval for ViV TAVR.

## 7. ViV TAVR Challenges

### 7.1. Coronary Obstruction

**Figure 4:**
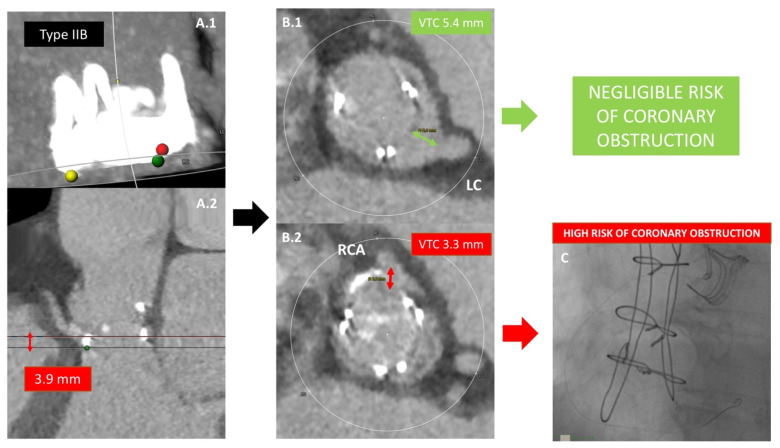
**Prediction of coronary artery obstruction**. A 60-year-old lady presented with Carpentier-Edwards Perimount BHV degeneration. The BHV frame extends above the coronary ostia, but below the sinotubular junction (**A.1**,**A.2**), showing RCA distance from the annulus of 3.9 mm). This situation is potentially at increased coronary obstruction risk. In this case, the following step is to calculate the VTC distance, intended as the distance between the prosthetic frame and coronary ostia: for the LCA the VTC is 5.4 mm (**B.1**) whereas a shorter VTC is depicted for the RCA (**B.2**). Considering a cut-off value of 4 mm, the patient is judged at negligible risk of LCA occlusion during ViV TAVR but at increased risk for RCA occlusion. For this reason, prophylactic RCA wiring and stenting are performed during ViV TAVR (**C**).

**Figure 5:**
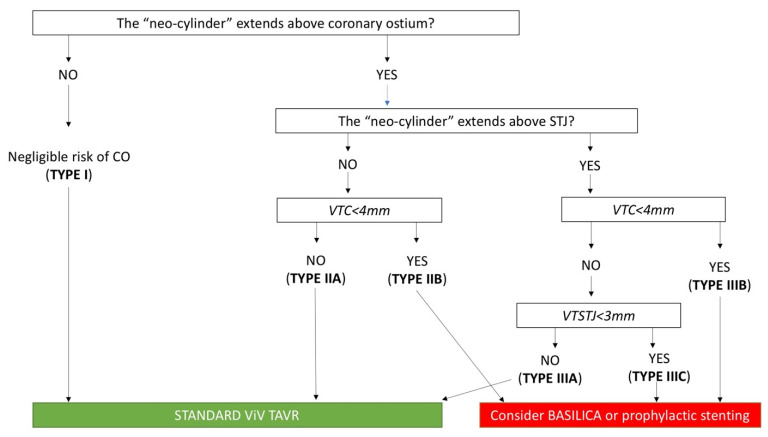
**Risk of coronary artery obstruction during ViV TAVR according to VIVID classification**. BASILICA: bioprosthetic aortic scallop intentional laceration to prevent iatrogenic coronary artery obstruction; CO: coronary obstruction; STJ: sinotubular junction; TAVR: trans-catheter aortic valve replacement; ViV: valve-in-valve; VTC: virtual-to-coronary distance; VTSTJ: virtual-to-sinotubular junction distance.

Coronary obstruction is a serious procedural complication, associated with a high mortality rate. This phenomenon is 3- to 4-fold more common after VIV TAVR when compared with native valve TAVR [31]. Its incidence was 3.5% in the VIVID Registry [32] and 2.5% in another multicenter registry [31]. After THV implantation, prosthetic leaflets are displaced in a tubular fashion from the circular frame to which they are attached, creating a cylinder effect. The “neo-cylinder” can come in direct, or near-direct, contact with the coronary ostium or the STJ and cause flow sealing off to the coronaries or sinus sequestration, respectively [33]. Predictors of coronary obstruction are dependent on the characteristics of the degenerated BHV and native aortic anatomy. In general, stentless BHVs and stented BHVs with externally mounted leaflets [e.g., Mitroflow (LivaNova PLC/Sorin Group, Saluggia, Italy) and Trifecta (Abbott, Minneapolis, MN, USA)] are at higher risk [34]; the formers due to the proximity of the leaflets to the usually re-implanted coronary arteries, the latter because the leaflets extend outward the BHV frame. Moreover, the presence of bulky bioprosthetic leaflets or a surgical BHV implanted in a supra-annular and/or slightly tilted position in regard to the long axis of the aortic root (reducing the distance between BHV and coronary ostia) can increase the risk of coronary obstruction. Among the patient’s anatomical features, the coronary ostia position above the upper edge of BHV frame minimizes the occurrence of coronary obstruction (type I of VIVID classification). The distance between the annulus and the coronary ostia (coronary artery height) is less relevant in ViV scenario if compared with native valve TAVR. In fact, the main predisposing factor is a short distance between the “neo-cylinder” and the coronary ostia or the STJ. These two distances can be reliable predicted at pre-procedural MSCT and have been known as VTC and VTSTJ (Figure 4). Low values are typically associated with narrow aortic root and a distance < 3–4 mm is considered at high risk for coronary obstruction. When the risk is prohibitive, bioprosthetic aortic scallop intentional laceration to prevent iatrogenic coronary artery obstruction (BASILICA) should be considered. However, BASILICA is challenging, requires a high level of technical skill, and can be associated with a non-negligible incidence of iatrogenic cerebral stroke [35]. Alternatively, a prophylactic coronary ostium stenting (Chimney technique) can be considered [36]. A comprehensive classification of aortic root anatomy in ViV TAVR and the consequently management algorithm has emerged from the VIVID Registry [37] (Figure 5).

### 7.2. Coronary Re-Access

Predictors of difficult coronary re-access after ViV TAVR are similar to those able to predict the risk of coronary obstruction. Bioprosthetic leaflets during ViV are tilted up, creating a “neo-skirt” that closes internally BHV frame struts. In this light, three aspects should be considered: the coronary ostia location in relation to the “neo-skirt”, the STJ dimensions, and the type and implantation mode of THV used. Briefly, coronary ostia can be situated above (sub-coronary risk plane) or below the upper edge (supra-coronary risk plane) of the BHV frame. The former scenario is associated with a predicted easy coronary artery re-cannulation, if a proper THV commissural alignment is performed. A supra-coronary risk plane is instead associated with an increased risk and the assessment of STJ dimensions is fundamental. A narrow STJ with a small VTSTJ distance (e.g., type IIa and IIIa of VIVID classification), beyond the increased risk of coronary obstruction, is associated with a more difficult coronary re-access given the absence of enough space for catheter manipulation. Lastly, the THV type plays a role in coronary re-access: short-frame BE-THVs and SE-THVs equipped with large upper stent frames/arches are associated with easier coronary re-cannulation. Engineering refinements have permitted to furnish the next-generation SE-THVs of different markers, that guide operators to perform a correct commissural alignment [38]. Positioning the prosthetic posts in-line with those of the surgical BHV, increases the chance for co-axial coronary artery re-cannulation. After TAVR-in-TAVR, coronary access may be challenging in a significant proportion of patients, as demonstrated by De Backer et al. on the basis of MSCT analyses: in this setting, THVs with intra-annular leaflet position or low commissural height and large open cells may be preferable in terms of coronary access after TAVR-in-TAVR [39].

### 7.3. Potential Post-Procedural Mismatch

One of the main issues related to ViV TAVR is the occurrence of post-procedural severe PPM, especially if the failed surgical BHV has a small true ID. It is important to remember that only for stented BHV with externally mounted leaflets the true ID overlaps the stent ID. For the other stented BHVs, the true ID is smaller compared to the stent ID: In porcine valves, the difference relies on 2 mm. In low surgical risk patients with a 19- or 21-mm BHV affected from SVD or severe PPM, repeat surgery should be advocated, with the caveat that the surgeon may need to perform a root enlargement so as to implant a large surgical valve size. In patients who are not candidates for surgery, then TAVR treatment for surgical BHV, compared with TAVR in native aortic valve disease, has demonstrated to have good durability and clinical outcomes at 3 years, despite higher gradients [15,16]. The use of an SE-THV with supra-annular design is generally preferred in this scenario, guaranteeing a better post-implantation hemodynamic profile. The drawback related to a small true ID and/or a pre-existent significant PPM [40], can be solved by BVF or be partially reduced by BVR. BVF and BVR are techniques to facilitate ViV TAVR, in which high-pressure balloon inflation is performed using a non-compliant balloon to either fracture the surgical valve ring or stretch the surgical valve ring or posts, allowing more optimal expansion of the THV. The ideal balloon size for BVF should always be more than the true ID of the failed BHV [6]. In a large multicenter series, BVF was safely performed in conjunction with both BE- and SE-THVs and resulted in significantly lower final transvalvular residual gradients and increased valve effective orifice area [41]. Recently, compared to ViV TAVR alone, ViV TAVR with BVF resulted in a significantly lower transvalvular gradient acutely and at follow-up [42]. In this study, independent predictors of lower gradients were the use of SE-THVs and the treatment of BHVs other than Mitroflow, irrespective of BVF performance; however, BVF significantly reduced the gradient independently from THV or surgical BHV type. The authors reported a 4% incidence of mechanical complications directly related to BVF. Nevertheless, the feasibility of BVF or BVR depends according to the type of failed BHV [43,44]. Moreover, some concerns still persist on the proper timing. If BVF is performed before TAVR, the advantage is easier implantation of THV with less sizing mismatch at the cost of a higher risk of hemodynamic collapse due to the occurrence of acute severe aortic regurgitation. Contrariwise, performing BVF after TAVR is associated with less risk of acute severe aortic regurgitation, but the disadvantages are a higher risk of device embolization and a potential THV leaflets injury. In conclusion, despite an overall good safety profile, the possibility of mechanical complications associated with BVF raises concerns about its risk-benefit ratio, particularly considering the lack of data about its potential beneficial clinical impact [17,45].

## 8. Knowledge Gaps

### 8.1. Stroke Risk and Cerebral Embolic Protection

Ischemic cerebral stroke is a rare, but fearsome, TAVR complication. Theoretically, heavily calcified and/or friable leaflets of a failed BHV can predispose to a higher risk of procedural ischemic stroke. A recent meta-analysis, including twenty-three studies, revealed that ViV TAVR is associated with a mean 30-day stroke and mortality rates of 2.2% and 4.2%, respectively [46]. However, no significant differences in 30-day stroke rate, 30-day mortality, and 1-year mortality between ViV TAVR and comparator treatment (native TAVR or redo surgical aortic valve replacement) were observed [46]. This finding makes the routine use of cerebral embolic protection device (CEPD) in ViV TAVR scenario still debatable. On the basis of the available evidence, CEPD should be positioned in selected cases, in which the risk of procedural stroke is increased due to several factors (e.g., patient’s clinical characteristics and/or the need to perform pre- and post-dilatation, BVF/BVR or BASILICA).

### 8.2. Antithrombotic Therapy

Recently, a consensus document provided by the ESC has shared light into the optimal antithrombotic regimen in patients undergoing TAVR [47]. Proposed recommendations encourage a minimalistic approach, preferring single antiplatelet therapy, whenever feasible. Nevertheless, the available evidence comes from studies in which the ViV TAVR subpopulation was excluded or under-represented [48,49,50]. RCTs addressing this topic are strongly needed.

## 9. Conclusions

ViV TAVR is a safe and effective treatment option for patients with failed BHVs, despite very long-term follow-up data are still scarce. Identifying the precise mechanism at the basis of device failure is pivotal to address the procedural feasibility. Multimodal imaging is crucial in both diagnostic and pre-procedural planning. The selection of THV depends on a multi-parametric assessment since special challenges are associated with ViV procedure. Coronary obstruction, severe PPM and subsequent difficult coronary re-access are at increased risk with ViV TAVR. For this reason, a detailed assessment and device selection are mandatory. Further RCTs are needed to address open hot topics in this scenario, such as the use of CEPD and the optimal post-procedural antithrombotic regimen.

## Figures and Tables

**Table 1:** Available evidence concerning ViV TAVR safety and efficacy.

First Author, Study	Year	Study Type	Type of Failed BHV	Comparator/Study Strategy	Patient Enrolled (*n*)	FU	Main Findings
Dvir D, VIVID [5]	2014	Retrospective, observational registry	Surgical BHV	-	459	1 year	- 30-day all-cause mortality: 7.6% - 30-day major stroke: 1.7% - 1-year all-cause mortality: 16.8%
Tuzcu EM, STS/ACC Registry [6]	2018	Retrospective, observational registry	Surgical BHV	TAVR in native valve/1:2 PSM	1150	1 year	- 30-day ViV TAVR all-cause mortality: 2.9% - For ViV TAVR lower 30-day mortality, 1-year mortality and HF re-hospitalization compared to TAVR for native valve
Webb, PARTNER 2 ViV Registry [7,13]	2017 and 2019	Prospective registry	Surgical BHV at high surgical redo risk	-	365	3 years	- 30-day all-cause mortality: 2.7% (0.7% for continued access patients) - 30-day CV death: 2.5% - 30-day all stroke: 2.7% - 30-day CO: 0.8% - 30-day PM implantation: 1.9% - 1-year all-cause mortality: 12.4% - 3-years all-cause mortality: 32.7%
Spaziano M [8]	2017	Retrospective study	Surgical BHV	Surgical redo/1:1 PSM	205 (78 pairs after PMS)	1 year	- Similar 30-day all-cause mortality (3.9% TAVR-in-BHV vs. 6.4% surgical redo, *p* = 0.49) - and 1-year all-cause mortality (12.3% vs. 13.1%, *p* = 0.80) - Similar 30-day stroke and PM implantation - Shorter hospitalization for TAVR-in-BHV
Tam DY [9]	2020	Retrospective, multicenter study	Surgical BHV	Surgical redo/1:1 PSM	558 (131 pairs after PSM)	5 years	- Lower 30-day all-cause mortality (ard: −7.5%), PPM implantation and blood transfusion for ViV TAVR - Higher 5-year survival for ViV TAVR (76.8% vs. 66.8%, *p* = 0.04)
Hirji SA [10]	2020	Retrospective study	Surgical BHV at high surgical redo risk	Surgical redo/1:1 PSM	6815 (2181 pairs after PSM)	30 day	- Unadjusted 30-day ViV TAVR all-cause mortality: 2.7% - Lower 30-day mortality (OR: 0.41), morbidity (OR: 0.72) and major bleeding (OR: 0.66)
Deharo P [11]	2020	Retrospective study	Surgical BHV	Surgical redo/1:1 PSM	717 pairs after PSM	516 days	- Lower 30-day composite endpoint of all-cause mortality, all-cause stroke, MI and major bleeding for ViV TAVR (OR: 0.62 *p* = 0.03) - No differences at long-term FU for composite endpoint of CV death, all-cause stroke, MI or HF re-hospitalization (OR: 1.18, *p* = 0.26)
Sá MPBO [12]	2021	Meta-analysis	Surgical BHV	Surgical redo	16207	30 days	- Lower 30-day all-cause mortality (OR: 0.53), stroke (OR: 0.65), PM implantation (OR: 0.73), major bleeding (OR: 0.49) for ViV TAVR - Higher 30-day MI (OR:1.50) and severe PPM (OR: 4.63) for ViV TAVR
Dauerman HL, CoreValve US Expanded Use Study [14]	2019	Prospective single-arm study	Surgical BHV at extreme surgical redo risk	-	226	3 years	- 3-year all-cause mortality or major stroke: 28.6%
Bleiziffer S [15], VIVID Registry long-term FU	2020	Retrospective observational registry	Surgical BHV	-	1006	3.9 years	- Estimated 8-year survival: 38%
Landes U [16]	2020	Retrospective observational registry	THV	-	212	1 year	- VARC-2 device success: 85.1% - 30-day all-cause mortality: 2.8% - 1-year all-cause mortality: 13.2%
Landes U [17]	2021	Retrospective observational registry	Failed THV and surgical BHV	TAVR-in-THV vs. TAVR-in-surgical BHV/1:1 PSM	1058 (165 pairs)	1 year	- Higher procedural success for TAVR-in-THV (72.7% vs. 62.4%, *p* = 0.045) - Similar procedural safety (70.3% vs. 72.1%, *p* = 0.715) - Similar 30-day (3% vs. 4.4%, *p* = 0.570) and 1-year (11.9% vs. 10.2%, *p* = 0.633) all-cause mortality

ACC: American College of Cardiology; ard: absolute reduction difference; BHV: bioprosthetic heart valve; CO: coronary obstruction; CV: cardiovascular; FU: follow-up; HF: heart failure; MI: myocardial infarction; OR: odd ratio; PARTNER: Placement of Aortic Trans-catheter Valves; PM: pacemaker; PPM: prosthesis-patient mismatch; PSM: propensity score matching; STS: Society of Thoracic Surgeons; TAVR: Trans-catheter aortic valve replacement; VARC: Valve Academy Research Consortium; ViV: valve-in-valve; VIVID: Valve-in-Valve International Data Registry.

**Table 2:** Feasibility of BVF/BVR in surgical BHVs.

BVF Feasibile	BVR Feasible	BVF/BVR Unfeasible
CE Magna CE Magna Ease CE Perimount 2800 and 2900 Mitroflow Mosaic Biocor Epic	Trifecta CE standard CE supra-annular Inspiris Resilia CE Perimount 2700	Hancock II Avalus Sutureless BHVs

BHV: bioprosthetic heart valve; BVF: balloon valve fracturing; BVR: balloon valve remodeling.

**Table 3:** SVD scale provided by EAPCI [23].

Stage	Echocardiographic Findings
0 (no SVD)	Normal valve morphology and function
1 (morphological SVD)	Intrinsic permanent structural changes to the prosthetic valve (leaflet integrity or structure abnormality, leaflet function abnormality, strut/frame abnormality)
2 (moderate haemodynamic SVD)	Mean transprosthetic gradient ≥ 20 mmHg and <40 mmHg Mean transprosthetic gradient ≥ 10 and <20 mmHg change from baseline Moderate intraprosthetic aortic regurgitation, new or worsening (>1 + /4) from baseline
Stage 3 (severe haemodynamic SVD)	Mean transprosthetic gradient ≥ 40 mmHg Mean transprosthetic gradient ≥ 20 mmHg change from baseline Severe intraprosthetic aortic regurgitation, new or worsening (>2 + /4) from baseline

EACPI: European Association of Percutaneous Coronary Intervention; SVD: structural valve deterioration.

## Abbreviations

BASILICA	Bioprosthetic aortic scallop intentional laceration to prevent iatrogenic coronary artery obstruction
BE	Balloon-expandable
BHV	Bioprosthetic heart valve
BVF	Balloon valve fracturing
BVR	Balloon valve remodeling
CEPD	Cerebral embolic protection device
EOA	Effective orifice area
ESC	European Society of Cardiology
ID	Inner diameter
MI	Myocardial infarction
MSCT	Multi-slice computed tomography
PPM	Patient-prosthesis mismatch
PVL	Paravalvular leak
RCT	Randomized controlled trial
SE	Self-expanding
STJ	Sinotubular junction
SVD	Structural valve deterioration
TAVR	Trans-catheter aortic valve replacement
THV	Trans-catheter aortic valve
ViV	Valve-in-valve
VARC	Valve Academic Research Consortium
VIVID	Valve-in-Valve International Data Registry
VTC	Virtual-to-coronary
VTSTJ	Virtual-to-sinotubular junction
